# On the surface effects of citrates on nano-apatites: evidence of a decreased hydrophilicity

**DOI:** 10.1038/s41598-017-09376-x

**Published:** 2017-08-21

**Authors:** Pavlo Ivanchenko, José Manuel Delgado-López, Michele Iafisco, Jaime Gómez-Morales, Anna Tampieri, Gianmario Martra, Yuriy Sakhno

**Affiliations:** 1 Department of Chemistry and Interdepartmental Centre “Nanostructured Interfaces and Surfaces-NIS”, University of Torino, Via P. Giuria 7, 10125 Torino, Italy; 2 Laboratorio de Estudios Cristalográficos, IACT (CSIC-UGR), Avda. Las Palmeras 4, E-18100 Armilla Granada, Spain; 3 Institute of Science and Technology for Ceramics (ISTEC), National Research Council (CNR), Via Granarolo 64, 48018 Faenza (RA), Italy

## Abstract

The surface structure and hydrophilicity of synthetic nanocrystalline apatite with strongly bound citrates on their surface are here investigated at the molecular level, by combining advanced IR spectroscopy, microgravimetry and adsorption microcalorimetry. Citrate are found to form unidentate-like and ionic-like complexes with surface Ca^2+^ ions, with a surface coverage closely resembling that present in bone apatite platelets (*i.e*., 1 molecule/(*n* nm)^2^, with *n* ranging between 1.4 and 1.6). These surface complexes are part of a hydrated non-apatitic surface layer with a sub-nanometre thickness. Noticeably, it is found that the hydrophilicity of the nanoparticles, measured in terms of adsorption of water molecules in the form of multilayers, decreases in a significant extent in relation to the presence of citrates, most likely because of the exposure toward the exterior of –CH_2_ groups. Our findings provide new insights on the surface properties of bio-inspired nano-apatites, which can be of great relevance for better understanding the role of citrate in determining important interfacial properties, such as hydrophobicity, of bone apatite platelets. The evaluation and comprehension of surface composition and structure is also of paramount interest to strictly control the functions of synthetic biomaterials, since their surface chemistry strongly affects the hosting tissue response.

## Introduction

Bio-inspiration is among the main concepts ruling innovation in the biomaterials field, where synthetic nanocrystalline apatite prepared in close to physiological conditions (so called “bio-inspired” or “biomimetic”) appears as a relevant subject, because of chemical composition and structure similar to that of the mineral phase present in human calcified tissues^1^. Indeed, apatite is present in bone and dentin as plate-shaped carbonated nanoparticles, ca. 30–50 nm in length as well as in width and with thicknesses ranging from 2 to 10 nm^2, 3^. Noteworthy, the peculiar shape and size of these biominerals are thought to be the result of a templating action carried out by the organic matrix driving complex biomineralization processes^4^. In particular, it was proposed that carboxylate-rich proteins like osteopontin, osteocalcin and amelogenin can significantly affect the crystallization pathway of apatite^5–7^. On this basis the effects of amino acids^8–10^, carboxylic acids^11, 12^ and peptides^13–15^ on nucleation, growth and facet stability of apatites nanoparticles were intensively investigated. In this scenario, citrates, the most abundant small molecules of the bone organic matrix (accounting for ~5.5 wt% of the organic matrix)^16^, seemed to play a minor role until the report of Schmidt-Rohr and co-workers^16^ which revealed that citrates are strongly bound on apatite nanocrystals in bone, stabilizing their size and morphology. In addition, Xie and Nancollas^17^ proposed that the use of this molecule could be a powerful nature-inspired strategy to finely tune the properties of synthetic apatite nanoparticles for biomedical applications. Davies *et al* ^18^. have also recently confirmed the presence of citrate bridging between bone apatite, which can explain the typical plate-like morphology of bone mineral.

The interaction between citrate and apatite surface apparently stemmed from the match between the spacing among −COO^−^ groups of citrates and that of Ca^2+^ ions exposed at the family of surfaces of highest morphological importance, namely the $\{10\overline{1}0\}$ one^16^. These surfaces were found to prevail by far also in the case of synthetic apatite crystallized in the presence of citrates^19, 20^. Depending on the preparation method, the amount of citrates strongly bound on the surface of some of these biomimetic apatite nanocrystals was similar to that found in bone apatites, i.e., 1 molecule/(2 nm)^2^ ^16^. This coverage corresponds to ca. 1/6 of the nanocrystal surface (considering that a citrate anion exhibits a projected geometrical area of 0.65 nm^2^)^21^. Thus, the features of the hybrid surface of these nanocrystals, mimicking the biogenic ones, should result from the combination of the presence of strongly bound citrates and the structure of the citrate-free portions around them.

The understanding of surface composition and structure is of paramount interest to tune material properties and functions, particularly in the case of biomaterials where surface is the boundary between the synthetic material and biomolecules of the hosting tissue^22^. This has been the aim of the research reported here, focused on the investigation of the impact of citrates on the surface interaction of apatite with water molecules, because of its relevance in ruling the interfacial behaviour towards biological media^22^. In fact, the presence of the –CH_2_ moieties of citrate was supposed to impart a local hydrophobicity to bone apatite nanocrystals, possibly relevant for the interaction of non-polar residues of collagen matrix^16, 23^. Here, an experimental assessment was targeted, by measuring the amount of adsorbed water molecules and the related adsorption energy on a set of citrate-hydroxyapatite nanoparticles matured over a time ranging from 4 to 96 hours (Cit−HA−Xh, with 4 ≤ × ≤ 96)^19, 24, 25^, in comparison with citrate-free hydroxyapatite (HA) nanoparticles overwhelmingly terminated by $\{10\overline{1}0\}$ planes^26^.

## Results and Discussion

### Structural, morphological and compositional features of Cit−HA

The understanding of the structural and morphological features of Cit−HA materials was the object of previous investigations^19, 24, 25^, and a summary of the most relevant results is listed in Supplementary Table S1. Briefly, morphologies and size distributions of crystal domains (hexagonal hydroxyapatite crystalline structure, space group *P6*_3_/m) of Cit−HA−4 h and Cit−HA−96 h were obtained from wide angle X-ray total scattering – Debye function analysis (WAXTS-DFA)^19^. To this aim, WAXTS patterns were modelled with two bivariate populations of Ca-deficient hydroxyapatite nanocrystals of increasing sizes with two different morphologies: hexagonal prisms (rod-like) and platy shapes (see Supplementary Fig. S1), in both cases elongated along the *c*-axis, and overwhelmingly limited by $\{\text{10}\mathop{\text{1}}\limits^{\bar{}}\text{0}\}$ planes^19^. Cit−HA−24 h was not included in that study, but it can be assumed that its structural features should fall within the findings obtained for the other two materials. The results showed an increase upon 4–96 h maturation in the mass fraction of crystals with hexagonal morphology *vs* the platy one from ∼43:57 to ∼59:41. Moreover, it was found that the average length and width of the crystalline domains (hexagonal fraction) vary from ∼21.0 to ∼25.1 nm and from ∼7.4 to ∼10.5 nm, respectively, upon maturation, while the length, width and thickness in the platy fraction varied from ∼21.0 to ∼25.1 nm, ∼8.9 to ∼12.4 and ∼3.9 to ∼5.4 nm, respectively. Overall, both morphologies exhibited a wide size distribution in length (standard deviation of the length dimension σ∼12.0 nm for the most matured sample) but a narrower distribution in width (σ∼2.5 nm) and thickness (σ∼1.1 nm).

The attachment of the crystals resulted in the formation of platy nanoparticles, with length, width and thickness, as measured by AFM, passing from (σ in brackets) 66.3(22.9) nm, 38.9(15.1) nm, 6.2(1.1) nm, respectively, to 104.1(19.0) nm, 56.6(12.2) nm, 13.5(4.1) nm over the maturation time considered^19^. The size of crystal domains and of nanoparticles was also measured in other works by conventional XRD and TEM, respectively^24, 25^. However, WAXTS appeared more informative on the structure of nanoparticles, because total scattering methods offer the unique advantage, compared to conventional diffraction, of treating Bragg scattering, originating from long-range order scattering, and diffuse scattering, originating from short-range effects, on an equal footing^19^. When comparing dimensions of nanoparticles as provided by TEM or AFM, the sizes of 2D projections observed in the first case are significantly affected by the arbitrary orientation of nanoparticles on the TEM grid. An assessment of the information provided by the various methods is reported in the Supplementary Discussion S1.

**Figure 1:**
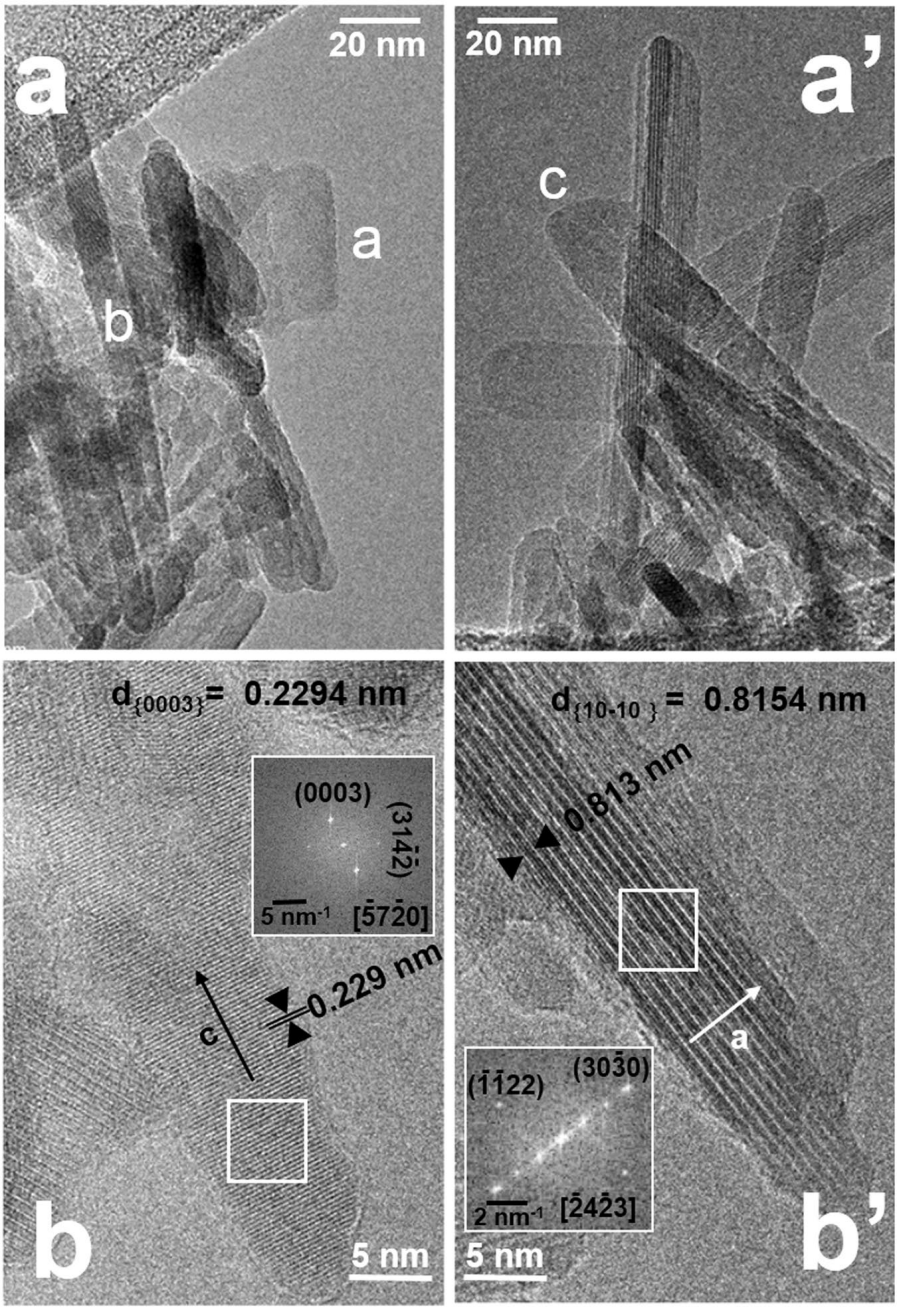
Representative TEM images of Cit-HA-24h. Original magnification: (**a,a**’) 150 k×; (**b,b**’) 400 k×. Insets in **b**,**b**’: FT of the image portions enframed within the white squares.

Here is worth mentioning that information complementary to AFM data can be obtained when the crystallographic orientation of nanoparticles can be retrieved by Fourier transform (FT) of HR−TEM images. An example is depicted in Fig. 1, where representative HR-TEM images of Cit−HA−24 h are shown, with the FT of selected zones.

At low magnification, different types of projected shapes can be observed (Fig. 1, panels **a**,**a’**), ranging from short plates (e.g. nanoparticle a, ca. 30 × 15 nm in size), to narrow rods (e.g. nanoparticle b, ca. 125 × 7 nm in size), to wider rods (e.g. nanoparticle c, ca. 80 × 20 nm in size). At high magnification, some nanoparticles exhibited lattice fringes, resulting from a statistical proper orientation with respect to the electron beam. For instance, lattice fringes due to {0003} planes oriented perpendicularly to the length of a projected shape were imaged (Fig. 1, panel b). The FT indicates that the particle was observed along the $[\overline{5}7\overline{2}0]$ direction (zone axis), with the *c*-axis in the image plane, i.e. corresponding to direction of elongation of the projected shape, and the *a*-axis pointing below the image plane with an angle of ∼45°. In another case, lattice fringes due to $\{10\overline{1}0\}$ planes appeared, parallel to the main side and borders of the particle projection (Fig. 1, panel b**’**). The zone axis resulting from the FT is $[\overline{2}4\overline{2}3]$, thus the *c*-axis is forming an angle of ∼20° below the image plane, while the *a*-axis lies in the image plane, i.e. it corresponds to the direction of the width of the projected shape.

WAXTS/DFA analysis also revealed that Cit−HA−4 h and Cit−HA−96 h contained a small fraction of an amorphous phase, which decreased upon maturation from ∼10% and 6% (mass fraction), respectively^19^. Neither in previous TEM/AFM investigations on Cit−HA materials matured for at least 4 h^19, 24, 25^, nor in the TEM observation of Cit−HA−24 h in the present work, amorphous calcium phosphate nanoparticles were observed. It can thus be inferred the amorphous phase should be along with the Cit-HA nanoparticles, likely located on the surface^19^. As a limit case, an amorphous layer thinner than 0.5 nm was theoretically estimated (see Supplementary Discussion S2).

**Figure 2:**
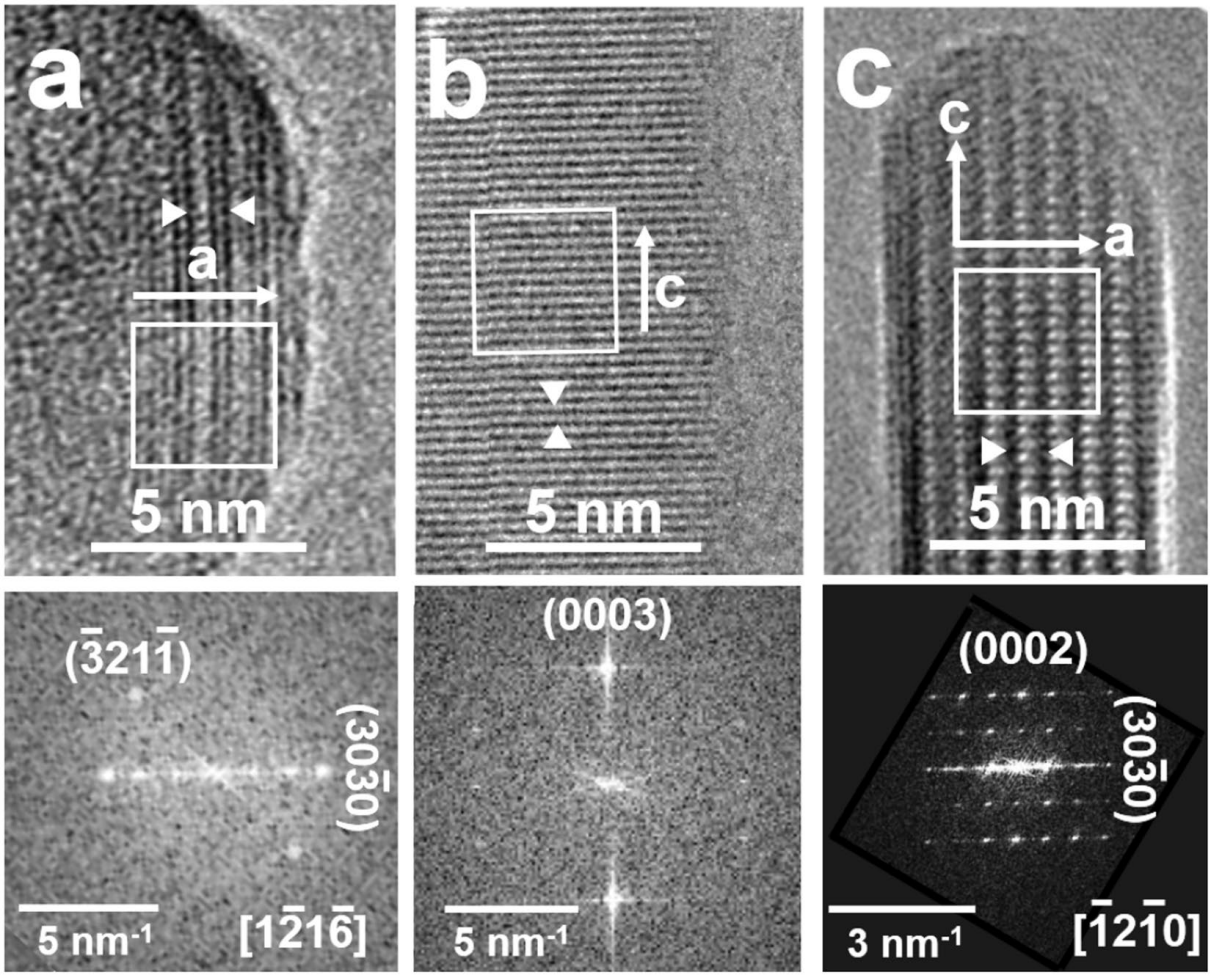
HR-TEM images of border regions of (**a**) Cit-HA-4 h, (**b**) Cit-HA-24 h and (**c**) Cit-HA-96 h. Interfringes spaces within the arrows are 0.814 nm in **a** and **c**, corresponding to ${{\rm{d}}}_{\{10\mathop{1}\limits^{\bar{}}0\}}$ = 0.8154 nm, and 0.229 nm in **b**, corresponding to d_{0003}_= 0.2294 nm. Original magnification: 400k ×. Insets: FT of the image portions enframed within the white squares. As indicated in the insets, particles were observed along the following directions (zone axes): (**a**) $[\overline{1}2\overline{1}6]$ (*a*-axis in the image plane, *c*-axis forming an angle of ∼55° below the image plane); (**c**) $[\overline{1}2\overline{1}0]$, i.e. the *b*-axis (*c-*axis in the image plane, *a*-axis forming an angle of 30° below the image plane).

Actually, a sub-nanometre amorphous layer can be observed by HR-TEM in some part of the borders of Cit−HA−4 h (Fig. 2a), whereas no evidence was obtained by this technique for Cit−HA−24 h and Cit−HA−96 h (Fig. 2b,c). Additional comments on the features of surface terminations are reported in the section devoted to surface hydration (*vide infra*).

Chemical analyses revealed that, irrespective of the precipitation time, Cit-HA nanoparticles were Ca-deficient of ca. 10% with respect to stoichiometric hydroxyapatite (Ca/P = 1.67) (Table 1, third column). It is important to remark that such Ca-deficiency is very close to that found for biological apatites, confirming the biomimetic nature of Cit-HA not only in terms of morphology and size but also in terms of chemical composition.

**Table 1:** Specific surface area (SSA_BET_), Ca/P ratio, citrate and carbonate content of samples synthesized in the absence (HA) and presence of citrate (cit-HA) at different maturation times. The citrate surface density is also provided, as both *n*_*cit*_/nm^2^and 1cit/(*n* nm)^2^.

Sample	SSA_BET_ (m^2^·g^−1^)	Ca/P^b^	Citrate content			Carbonate content (wt%)
			(wt%)	Surface density		
				*n*_*cit*_/nm^2^	1cit/(*n* nm)^2^	
HA1^a^	130 ± 6		0	0	0	1.0 ± 0.1
HA2^a^	160 ± 10		0	0	0	1.8 ± 0.2
Cit–HA–4 h	160 ± 10	1.51 ± 0.02	2.4 ± 0.1^b^	0.47 ± 0.1	1.46 ± 0.3	1.1 ± 0.1^b^
Cit–HA–24 h	145 ± 7	1.52 ± 0.03	1.9 ± 0.1^b^	0.41 ± 0.1	1.56 ± 0.4	1.0 ± 0.1^b^
Cit–HA–96 h	114 ± 5	1.54 ± 0.03	2.0 ± 0.1^b^	0.55 ± 0.1	1.35 ± 0.2	1.0 ± 0.1^b^

^a^Citrate-free hydroxyapatite studied in ref. 26.

^b^ref. 25.

Residual amounts of carbonate were present in Cit-HA nanoparticles irrespective of the maturation time (Table 1), being close to 1 wt%^25^. However, their IR-ATR spectra did not exhibit any observable feature in the 800–900 cm^−1^ (Supplementary Fig. S2) related to A- or B-type carbonate substitutions, confirming that the actual carbonate content is residual. The amount of citrate did not change with the maturation time, while the SSA_BET_ decreased (Table 1), which is in agreement with the observed size increase upon maturation (see above and Supplementary Table S1).

### Citrate-Ca^2+^ interaction

**Figure 3:**
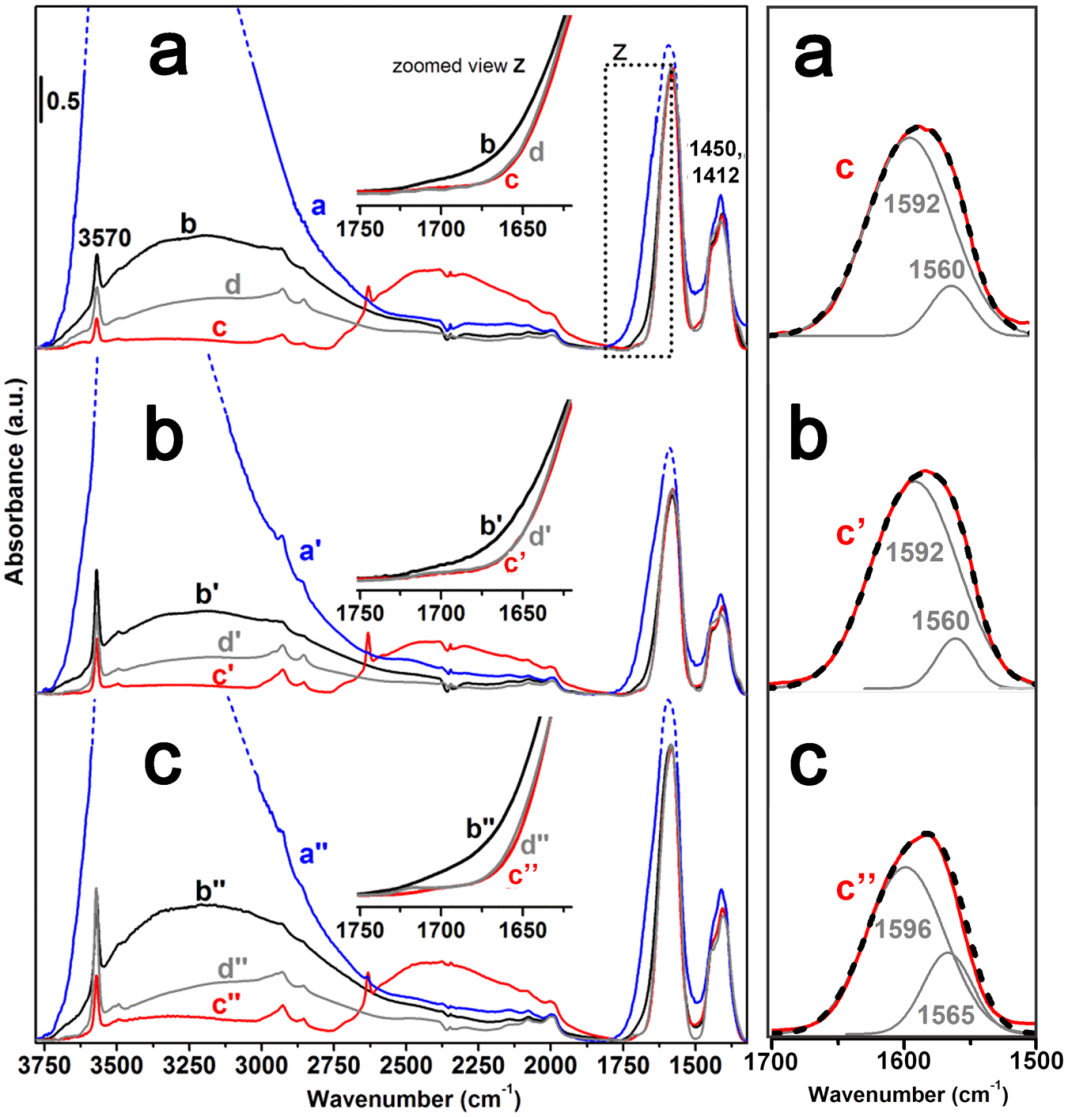
IR spectra of (**a**) Cit–HA–4 h, (**b**) Cit–HA–24 h; (**c**) Cit–HA–96 h. Left panel, curve labelling in left panel is: (**a,a**’**,a**”) in contact with H_2_O vapor at 20 mbar; (**b,b**’**,b**”) after 60 min outgassing at b.t.; (**c,c**’**,c**”) after exchange with D_2_O and subsequent 60 min outgassing at b.t.; (**d,d**,’**d**”) after back exchange with H_2_O and subsequent 120 min outgassing at 433 K. Inset: zoomed view of the 1750–1600 cm^−1^ range. Right panel: zoomed view of the 1700–1500 cm^−1^ range, showing the experimental νCOO^−^_asym_ band in curves **c,c**’**,c**” (thus without any δH_2_O contribution) and related fitting (dashed black curve = sum of individual components, in grey).

Insights on the coordination of citrates to surface Ca^2+^ were obtained by the analysis of the IR spectra of the three Cit−HA materials. In Fig. 3, curves (a, a’, a”) are the spectra of specimens in contact with H_2_O vapor at 20 mbar (assignment of signals in the 3800–1800 cm^−1^ range in Table 2), resulting in the formation of adsorbed water multilayers^26–29^. Samples were then outgassed at beam temperature (hereafter b.t.) (curves b, b’, b”). This treatment left adsorbed only water molecules coordinated to surface Ca^2+^ ions^26–29^, and, if the case, in subsurface position in hydrated non-apatitic layers^25, 30^.

**Table 2:** General assignment of bands present in the 3800−1800 cm^−1^ range of the IR spectra of Cit−HA−4 h, Cit−HA−24 h; Cit−HA−96 h in contact with 20 mbar of H_2_O, after 60 min outgassing at b.t., and after exchange with D_2_O and subsequent outgassing at b.t.

Band position (cm^−1^)	Assignment^28, 29^
3750−2500 (range)	Stretching modes of adsorbed H_2_O, bulk and surface hydroxy groups (e.g., of HPO_4_^2−^) involved in H-bonding
3570 (maximum)	Stretching mode of bulk OH^−^, occupying the 4e position in the hexagonal lattice (so-called “columnar OH”, since they are aligned in columns parallel to the *c*-axis)
2750−2000 (range)	Stretching modes of adsorbed D_2_O, and surface −OD groups involved in D-bonding
2635 (maximum)	Stretching mode of bulk “columnar” OD^−^
2200−1800 (range)	Combination modes of fundamental phosphate bulk absorption (falling below the transparency limit in the adopted experimental conditions)

Focusing on the spectral range below 2000 cm^−1^, the comparison between the spectra of materials outgassed at b.t. in their pristine form (curves b, b’, b”) and after H/D exchange by contact with D_2_O (curves c, c’, c”) indicates that the deformation mode of water molecules (δH_2_O) contributes as a weak, ill resolved shoulder on the high frequency side of the main band at 1585 cm^−1^, as it disappears after isotopic exchange (Fig. 3, left panel, insets “z”; the δD_2_O signal at ca. 1220 cm^−1^ falls below the lower limit of the spectral range shown). It should be noted that this shoulder was no longer present after outgassing at 433 K (curves d, d’, d”), indicating that H_2_O molecules were completely removed by this treatment.

As reported above, carbonate ions are present in a substantially low amount, as such they are expected not to contribute significantly to the spectral pattern in the 1650–1350 cm^−1^ range, where some of their typical signal falls^31^. Thus, the main component at 1580 cm^−1^ is attributed to the antisymmetric (asym) stretching of −COO^−^ groups, the symmetric (sym) partner mode producing the pattern in the 1500 – 1350 cm^−1^ range^11, 32–34^. It is worth mentioning that both these components were affected in a very limited extent by the presence/absence of water molecules adsorbed in multilayers (curves a, a’, a”, and b, b’, b”, respectively; in the first set of curves, νCOO^−^_asym_ bands are partly overlapped with the δH_2_O signal). This behaviour indicated that all carboxylate groups of citrate molecules should interact quite strongly with the apatitic surface. Nevertheless, citrates should be overwhelmingly located at/near the surface of nanoparticles, because washing with a basic solution removed ca. 90% of them (see Supplementary Fig. S3), without significant changes to neither SSA_BET_ nor XRD patterns (see Supplementary Fig. S4). For all Cit−HA materials, the νCOO^−^_asym_ signal was properly fitted by using two components (with a small difference in position for Cit−HA−96 h with respect to the other two samples), with the sub−band located at higher frequency exhibiting the highest intensity (Fig. 3, right panel). The presence of these two sub−bands has a counterpart in the two components at 1450 and 1412 cm^−1^ in the νCOO^−^_sym_ pattern. This indicates the occurrence of two different types of interaction of −COO^−^ groups with their surroundings. This finding is in good agreement with the presence of two −COO^−^·apatite surface distances found by REDOR NMR in native bone with a similar coverage of strongly bounded citrates^16^.

The ensemble of data reported above suggests that in the considered Cit−HA materials citrates might mainly exhibit an orientation allowing all three −COO^−^ groups of a citrate molecule to interact with the surface. Very recently, Wang *et al*.^20^ calculated that “standing up” (interacting with the apatitic surface through only one −COO^−^ group) and “lying down” conformations of citrates on the $\{\text{10}\mathop{\text{1}}\limits^{\bar{}}\text{0}\}$ apatite surface should be isoexergonic. However, these authors stated that the “lying down” conformation should prevail at low citrate coverage (as that of the HA-Cit materials considered in this work, ca. 0.5 molecule nm^−2^), while the experimental evidence of the coexistence of “lying down” and “standing up” conformations was obtained for citrate coverage in the 2.3–4.0 molecule nm^−2^ range.

The splitting (Δν) between asym and sym modes frequencies of a carboxylate coordinating a cationic centre depends on the type of complexing, namely unidentate, bidentate (chelating), bridging or ionic^35^. The present data do not provide specific information on actual asym-sym band pairing, although some insights can be extracted after analysing the four possible combinations:

$$\begin{array}{c}-1592/1596\,{\rm{a}}{\rm{n}}{\rm{d}}\,1412\,{{\rm{c}}{\rm{m}}}^{-1}\,{\rm{c}}{\rm{o}}{\rm{m}}{\rm{p}}{\rm{o}}{\rm{n}}{\rm{e}}{\rm{n}}{\rm{t}}{\rm{s}}:{\rm{\Delta }}\nu =180/184\,{{\rm{c}}{\rm{m}}}^{-1}\\ -1592/1596\,{\rm{a}}{\rm{n}}{\rm{d}}\,1450\,{{\rm{c}}{\rm{m}}}^{-1}\,{\rm{c}}{\rm{o}}{\rm{m}}{\rm{p}}{\rm{o}}{\rm{n}}{\rm{e}}{\rm{n}}{\rm{t}}{\rm{s}}:{\rm{\Delta }}\nu =142/146\,{{\rm{c}}{\rm{m}}}^{-1}\\ -1560/1565\,{\rm{a}}{\rm{n}}{\rm{d}}\,1412\,{{\rm{c}}{\rm{m}}}^{-1}\,{\rm{c}}{\rm{o}}{\rm{m}}{\rm{p}}{\rm{o}}{\rm{n}}{\rm{e}}{\rm{n}}{\rm{t}}{\rm{s}}:{\rm{\Delta }}\nu =148/153\,{{\rm{c}}{\rm{m}}}^{-1}\\ -\,1560/1565\,{\rm{a}}{\rm{n}}{\rm{d}}\,1450\,{{\rm{c}}{\rm{m}}}^{-1}{\rm{c}}{\rm{o}}{\rm{m}}{\rm{p}}{\rm{o}}{\rm{n}}{\rm{e}}{\rm{n}}{\rm{t}}{\rm{s}}:{\rm{\Delta }}\nu =110/115\,{{\rm{c}}{\rm{m}}}^{-1}\end{array}$$

Second and third combinations result in very similar Δν values, thus they should correspond to carboxylate complexes that are similar in structure. Thus, it is unlikely they account for the presence of two pairs of νCOO^−^_asym_ and νCOO^−^_sym_ bands. For the first and fourth combinations, the splitting must be compared with the Δν = 115 cm^−1^ appearing in the spectrum of calcium citrate ionic salt^36^. The largest Δν = 180/184 cm^−1^ can monitor the presence of unidentate-like complexes, whilst the smallest Δν = 110/115 cm^−1^ can be attributed to −COO^−^ groups forming ionic-like complexes with surface Ca^2+^ sites^35^. On the basis of the relative intensity of the two band pairs, unidentate-like complexes will be the more abundant.

The proposed coordination structures are different from the chelating ones proposed by Achelhi *et al*.^11^, however their assignment was based on the comparison of the Δν of citrates on hydroxyapatites with the Δν exhibited by other carboxylate species, but not by considering the usual reference, i.e. the relevant ionic form^35^. It is also worth mentioning that the similarity in position of the spectral pattern due to carboxylates indicates that the coordination of citrates to Ca^2+^ ions did not change significantly upon maturation.

### Surface hydration

The subsequent step of the investigation of the surface properties of Cit−HA materials was aimed at quantifying the surface hydration and at evaluating the corresponding energetic aspects of the water-surface interactions. The amounts of water molecules left adsorbed after outgassing at 323 K and adsorbed by samples in contact with 20 mbar of water vapor are listed in Table 3. Previous studies proved that ca. 1 H_2_O per surface cationic site is left adsorbed by outgassing nano-sized hydroxyapatites at the indicated temperature^26, 28, 29^. In the case of $\{\text{10}\mathop{\text{1}}\limits^{\bar{}}\text{0}\}$ surfaces of stoichiometric hydroxyapatite (Ca/P = 1.67) this so called “first hydration layer” corresponds to ca. 4.5 H_2_O·nm^26, 28, 29^. A similar value was found for the first hydration layer on citrate-free HA1 and HA2 (Table 3, column 2), exhibiting a Ca/P ratio slightly lower than 1.67 (see Table 1) and overwhelmingly terminated by $\{\text{10}\mathop{\text{1}}\limits^{\bar{}}\text{0}\}$ surfaces^26^.

**Table 3:** Water surface density, ${n}_{{H}_{2}O}$ (given as water molecules per nm^2^) for the two hydration conditions considered.

Materials	${{\boldsymbol{n}}}_{{{\boldsymbol{H}}}_{{\boldsymbol{2}}}{\boldsymbol{O}}}$, (fraction irreversible by outgassing at 323 K)	${{\boldsymbol{n}}}_{{{\boldsymbol{H}}}_{{\boldsymbol{2}}}{\boldsymbol{O}}}$, (fraction reversible by outgassing at 323 K)
HA1	4.4 ± 0.5	8.2 ± 0.7
HA2	4.1 ± 0.5	8.4 ± 0.7
Cit–HA–4 h	7.9 ± 0.7	6.3 ± 0.6
Cit–HA–24 h	3.1 ± 0.4	5.8 ± 0.6
Cit–HA–96 h	3.5 ± 0.4	4.6 ± 0.5

Conversely, the amount of H_2_O per nm^2^ still presents on Cit−HA−4 h after outgassing at b.t. exceeded the maximum density of water molecules coordinated to cations exposed on $\{\text{10}\mathop{\text{1}}\limits^{\bar{}}\text{0}\}$ surfaces. Moreover, it must be considered that H_2_O molecules left on Cit-HA-4h by outgassing at b.t. appeared all sensitive to the H/D isotopic exchange by adsorption/desorption of D_2_O (Fig. 3 and related comments), thus they are not entrapped in the bulk of nanoparticles. Both these features are consistent with the supposed presence of a non-apatitic, amorphous surface layer, significantly hydrated (i.e. with H_2_O molecules also in sub-surface position, accessible for isotopic exchange), with citrates mainly located at/near its exterior termination. The existence of hydrated surface terminations of a similar type (without citrates) was reported by Rey *et al*. for biomimetic nanocrystalline apatites prepared in a different way^30^. For Cit−HA−4 h, the amount of H_2_O molecules in this layer, expressed in terms of apparent density per surface unit (Table 3), appeared ca. double with respect to the first hydration layer expected for a stoichiometric $\{\text{10}\mathop{\text{1}}\limits^{\bar{}}\text{0}\}$ surface, suggesting the layer should be very thin, in agreement with the calculated thickness of the surface amorphous layer (see above) and HR-TEM observations (Fig. 2a).

In the case of Cit−HA−24 h and Cit−HA−96 h, the amount of water left adsorbed after outgassing at 323 K decreased significantly, indicating that maturation resulted also in a dehydration of surface layers. Nevertheless, this dehydration did not affect the coordination structure of citrates to surface Ca^2+^ ions (see Fig. 3 and related comments).

The density of water molecules considered as located only at the surface of nanoparticles of these two materials resulted in ca. 3.0–3.5 H_2_O/nm^2^, thus lower than for citrate-free nano-HA. This decrease is consistent with both the Ca^2+^ deficiency of Cit−HA materials and the occupancy of surface cations by −COO^−^ of citrates, in reason of ∼1.23–1.65 per nm^2^_,_ as results from the surface density of citrates ranging from ∼0.41 to ∼0.55 cit^3−^ per nm^2^ (Table 1) and the coordination of each −COO^−^ to a Ca^2+^ site inferred from IR data (Fig. 3).

The supposed presence of an outmost amorphous layer and the lack of relevant insights on its content in Ca^2+^ prevented the possibility to evaluate if the water molecules in the primary hydration layer of Cit−HA−24 h and Cit−HA−96 h are only coordinated to the surfaces or also in sub-surface positions.

Despite the complexity and related incertitude on the actual atomistic surface structure of Cit-HA materials, significant insights on their hydrophilicity evaluated in respect to citrate-free HA resulted from the measurement of the amount of water molecules adsorbed over the first surface molecular layer constituted by only water molecules (for HA1 and HA2) or H_2_O and cit^3−^ (for the Cit-HA samples). As reported in the last column of Table 3, the presence of citrates decreases in a significant extent the capability to adsorb water, and thus the hydrophilicity of Cit-HA materials. This behaviour can be easily associated to the exposure towards the exterior of −CH_2_ groups of citrates bound to surface Ca^2+^ ions through their −COO^−^ moieties. This finding appears as the evidence of what was proposed by Schmidt-Rohr and co-workers on the basis of the orientation of citrates on apatite nanocrystals in bone^16^.

**Figure 4:**
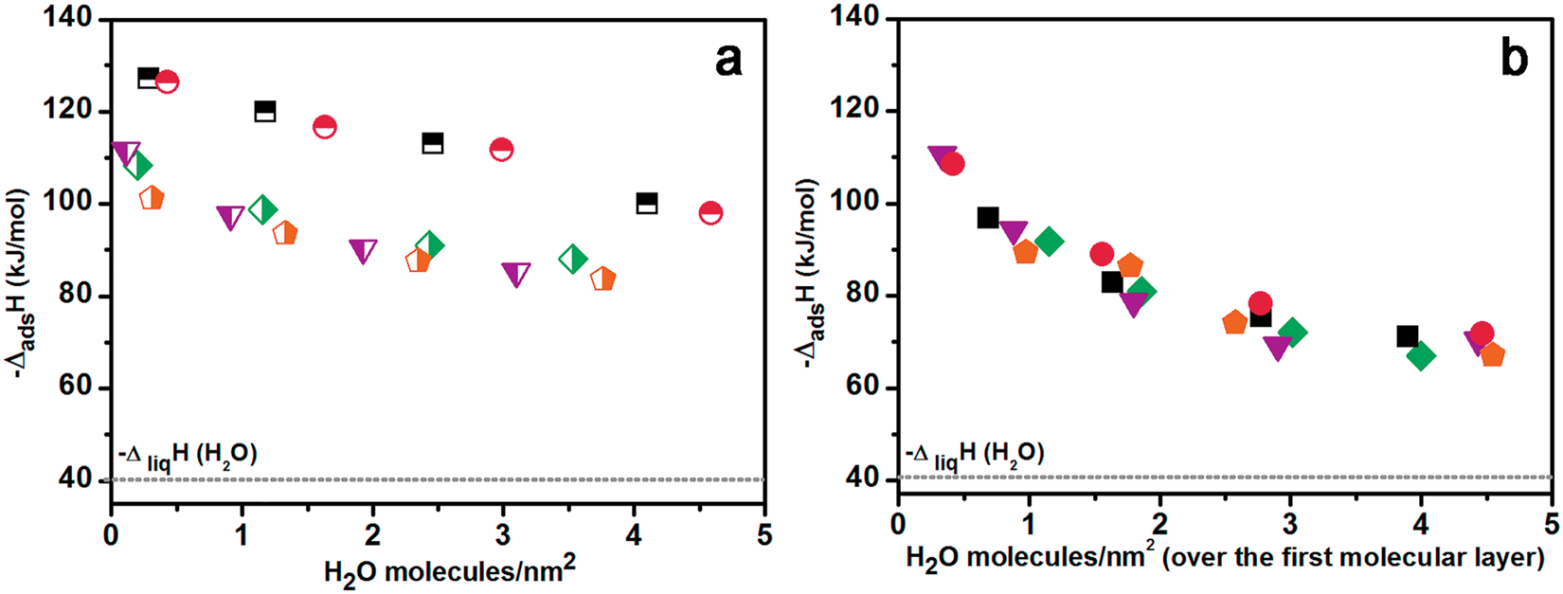
Enthalpy of adsorption versus uptake of H_2_O molecules on: **red** symbols = HA1; **black** symbols = HA2; **orange** symbols = Cit–HA–4 h; **purple** symbols = Cit–HA–24 h and **green** symbols = Cit–HA–96 h. Panel a, half-filled symbols: attainment of the first hydration layer. Panel b, full symbols: additional adsorption of water, within the limit of the method. Related volumetric isotherms (adsorbed H_2_O molecules·nm^2^ *vs*. pH_2_O) are in Supplementary Fig. S5. The dotted line indicated the value of latent enthalpy of liquefaction of water, −Δ_liq_H(H_2_O), for the sake of comparison.

Energetic aspects of the H_2_O−surface interaction were investigated by adsorption microcalorimetry. Adsorption enthalpy (−Δ_ads_H) measured by admitting water on the three Cit−HA materials outgassed at 433 K, thus exposing fully dehydrated Ca^2+^ ions (Fig. 3 and related comments) are reported in Fig. 4a, in comparison with analogous data obtained for citrate-free HA1 and HA2 materials.

These data, dealing with the attainment of the first hydration layer, appeared grouped in two families: one water on citrate-free HA1 and HA2 materials, with ca. 130 ≥ −Δ_ads_H ≥ 100 kJ·mol^−1^, whilst values obtained for Cit−HA materials appeared ca. 20 kJ·mol^−1^ lower. Nevertheless, these latter values were still high enough (ca. 110 ≥ −Δ_ads_H ≥ 90 kJ·mol^−1^) to monitor the occurrence of a relatively strong interaction between water molecules and surface sites^37^.

This overall downshift of −Δ_ads_H curve for Cit-HA samples indicates the local structure of Ca^2+^ surface sites of these materials resulting from the dehydration at 433 K should be different with respect to surface termination of HA nanoparticles grown in the absence of citrates. This feature can result by short-range inductive effects of citrates on neighbouring cationic sites and/or by the transformation of pristine non-apatitic hydrated surface layers during water removal.

However, the impact on the energy of water adsorption appears to be limited to the formation of the first hydration layer. Indeed, the enthalpy of the further adsorption of water (within the limit of the method, see the Experimental section) appeared almost coincident for citrate−free and Cit–HA materials (Fig. 4b). In all cases, the interaction among water molecules resulted stronger by far than for liquid water (latent enthalpy of liquefaction of water, −Δ_liq_H(H_2_O) = 44 kJ·mol^−1^). This was interpreted as an indication that H_2_O molecules coordinated to surface Ca^2+^ sites of nano-apatites are strongly polarized by the interaction with the cation sites, and in turn exhibit a strong affinity towards water^37^.

## Conclusion

The investigation at the molecular level carried out in this work reveals that citrates strongly bound to the surface of the nano-apatites, with each −COO^−^ forming unidentate-like and/or ionic-like complexes with surface Ca^2+^ ions. After 4 h of maturation, citrate-Ca^2+^ complexes are part of a hydrated non-apatitic surface layer exhibiting a sub-nanometer thickness, which become thinner and undergoes a significant dehydration upon further maturation. Conversely, the citrate-Ca^2+^ bonding configurations are kept unchanged (at least for the time interval considered in this work).

Noteworthy, strongly bound citrates affect interfacial behaviour toward water, significantly decreasing the surface hydrophilicity, here measured as adsorbed H_2_O molecules multilayers, likely because of the exposure toward the exterior of their −CH_2_ groups.

## Methods

### Materials

Calcium chloride dihydrate (CaCl_2_·2H_2_O, Bioxtra, P99.0% pure), sodium citrate tribasic dihydrate [Na_3_Cit·2H_2_O, ACS reagent, ≥99.0% pure, where Cit: C_6_H_5_O_7_], sodium phosphate dibasic (Na_2_HPO_4_, ACS reagent, ≥99.0% pure), sodium hydroxide (NaOH, ACS reagent, ≥97.0%) were supplied by Sigma–Aldrich. All the solutions were prepared in ultrapure water (0.22 μS, 298 K, MilliQ, Millipore).

Cit-HA nanoparticles were precipitated using the thermal-decomplexing batch method, as previously described^25^. Briefly, a solution containing 0.1 M CaCl_2_ and 0.4 M Na_3_Cit was poured to a solution containing 0.12 M Na_2_HPO_4_ at room temperature. The pH of the mixture was adjusted to 8.5 with HCl and then maturated at 353 K for 4 (material code: Cit−HA−4 h), 24 (material code: Cit−HA−24 h) and 96 (material code: Cit−HA−96 h) hours. The precipitates were then washed repeatedly with MilliQ water by centrifugation and finally freeze-dried overnight at 223 K. Final citrate content of samples are listed in Table 2.

With the aim of removing citrate ions contained in the Cit-HA samples, 10 mg of the materials were washed three times with 10 ml of NaOH 0.1 M by centrifugation, finally washed with MilliQ water and freeze-dried overnight at 223 K.

For the sake of comparison, two types of citrate-free nano-apatites were also considered, hereafter referred to as HA1 and HA2. They were the subjects of a previous study^26^, where full details on their preparations are reported. In summary, the preparation conditions were:

- HA1: a solution of H_3_PO_4_ was dropped in a Ca(OH)_2_ suspension (both 1.35 M). The reaction mixture was stirred overnight at ca. 310 K. Subsequently, the mixture was left standing for 2 h to allow for deposition of the inorganic phase, which was then isolated by centrifugation of the mother liquor, repeatedly washed with water and dried at 213 K under outgassing (residual pressure: 3 mbar) overnight.
- HA2: the overall procedure was the same, but the starting reagents were a H_3_PO_4_ solution (0.21 M) and a Ca(CH_3_COO)_2_ solution (0.35 M), and pH was maintained at ca. 10 by addition of a NH_4_OH solution.

For the investigation of surface hydration, H_2_O (MilliQ) and D_2_O (99.9 atom % D, Aldrich) were admitted onto the samples after several freeze-pump-thaw cycles. For IR measurements, high-purity CO (Praxair) was employed without any additional purification except liquid nitrogen trapping.

### Characterisation Methods

Specific surface area (SSA_BET_) was measured with a Micromeritics ASAP 2010 by nitrogen adsorption at 77 K, using the BET model for data treatment. Materials were outgassed at 300 K for 10 h, i.e. until the attainment of a residual pressure of 1 × 10^−3^ mbar. The same procedure was adopted for samples otherwise outgassed at 433 K for other measurements, then recovered and re-exposed to air.

High-resolution transmission electron microscopy (HR-TEM) images of Cit-HA-24h were taken with a JEOL 3010 instrument, operated at 300 kV. Images of the other materials were already acquired for previous works (HA1 and HA-2: ref. 26. Cit-HA-4h and Cit-HA-96-h: ref. 24). For the observation, the sample, in the form of fine powder, was contacted with a Cu grid coated with a lacey carbon film. Feeble illumination conditions were adopted during the observation to avoid any modification of the material (being apatites quite sensitive to inelastic scattering by the electron beam)^38^.

For microgravimetric measurements, carried out in triplicate with a Hiden Intelligent Gravimetric Analyzer IGA002 instrument, the materials were initially compacted in self-supporting pellets, subsequently broken in several pieces, in order to avoid loss of ultrafine particles induced by possible turbulence in the sample holder during desorption steps. In a first series of experiments, samples were outgassed at 323 K (residual pressure p ≤ 10^−4^ mbar) and then contacted with 23.5 mbar of H_2_O vapour at the same temperature. In a second one, the outgassing temperature was increase up to 433 K (*vide infra*), cooled down to 323 K and then contacted with the same H_2_O vapour pressure. Subsequently, an additional H_2_O desorption/adsorption sequence at 323 K was performed.

### Adsorption Microcalorimetry

The volumetric−calorimetric setup allowed all thermal treatments in vacuo as well as adsorption–desorption experiments to be carried out *in situ*.

Prior to the adsorption experiments, the samples (pieces of broken self-supporting pellets) were outgassed at T = 433 K for 60 min (residual pressure p ≤ 10^−4^ mbar). The enthalpy change associated with the adsorption was measured at T = 303 K by means of a heat-flow microcalorimeter (Calvet C80, Setaram, F) connected to a homemade, high-vacuum gas-volumetric glass apparatus (residual pressure p ≤ 10^−4^ mbar). A well-established stepwise procedure was followed^39^, which allowed to determine, during the same experiment and for subsequent small increments of the adsorptive, both adsorbed amounts and integral heats evolved, as a function of the increasing equilibrium pressure. This latter was monitored by a transducer gauge (Ceramicell 0–133,33 mbar, Varian). The maximum H_2_O pressure in equilibrium with the samples was limited to 10 mbar, a conservative value for an effective use of the perfect gas law for data analysis.

The calorimetric outputs (integral heats evolved during the adsorption, Q^int^) were routinely processed to obtain the differential heats of adsorption (q_diff_ = −Δ_ads_H, kJ mol^−o^), which quantify with a reasonable accuracy the energy of interaction of the molecular probe with the individual adsorption sites.

## Electronic supplementary material

Supplementary Information

## References

[1] Wegst UGK, Bai H, Saiz E, Tomsia AP, Ritchie RO (2015). Bioinspired structural materials. Nat. Mater..

[2] Eichert, C., Drouet, C., Sfihi, H., Rey, C. & Combes, C. Nanocrystalline apatite-based biomaterials: synthesis, processing and characterization. in Biomaterials Research Advances(ed Jason B. Kendall) 91–191 (Nova Science Publisher, 2007).

[3] Lowenstam, H. A. & Weiner, S. On Biomineralization. 336 (Oxford University Press, 1989).

[4] Nudelman F, Sommerdijk NAJM (2012). Biomineralization as an inspiration for materials chemistry. Angew. Chem. Int. Ed..

[5] Deshpande AS, Fang PA, Simmer JP, Margolis HC, Beniash E (2010). Amelogenin-collagen interactions regulate calcium phosphate mineralization *in vitro*. J. Biol. Chem..

[6] Hunter GK, Kyle CL, Goldberg HA (1994). Modulation of crystal-formation by bone phosphoproteins - structural specificity of the osteopontin-mediated inhibition of hydroxyapatite formation. Biochem. J..

[7] Yang Y, Cui QA, Sahai N (2010). How does bone sialoprotein promote the nucleation of hydroxyapatite? A molecular dynamics study using model peptides of different conformations. Langmuir.

[8] Boanini E, Torricelli P, Gazzano M, Giardino R, Bigi A (2006). Nanocomposites of hydroxyapatite with aspartic acid and glutamic acid and their interaction with osteoblast-like cells. Biomaterials.

[9] Jahromi, M. T., Yao, G. & Cerruti, M. The importance of amino acid interactions in the crystallization of hydroxyapatite. *J. R. Soc. Interface* **10**, (2013). 10.1098/rsif.2012.0906 PMC3565740 23269851

[10] Palazzo B (2009). Amino acid synergetic effect on structure, morphology and surface properties of biomimetic apatite nanocrystals. Acta Biomater..

[11] Achelhi K (2010). Role of carboxylate chelating agents on the chemical, structural and textural properties of hydroxyapatite. Dalton Trans..

[12] Shimizu H, Zhuang Z, Aizawa M (2013). Morphological control of hydroxyapatite particles by homogeneous precipitation method in the co-presence of various carboxylic acids. Bioceramics.

[13] Long JR (1998). A peptide that inhibits hydroxyapatite growth is in an extended conformation on the crystal surface. Proc. Natl. Acad. Sci. USA.

[14] Wang JJ (2015). Biomimetic synthesis of platelet-shaped hydroxyapatite mesocrystals in a collagen mimetic peptide-PEG hybrid hydrogel. Mater. Lett..

[15] Xiao Y (2015). Hydroxyapatite growth inhibition effect of pellicle statherin peptides. J. Dent. Res..

[16] Hu YY, Rawal A, Schmidt-Rohr K (2010). Strongly bound citrate stabilizes the apatite nanocrystals in bone. Proc. Natl. Acad. Sci. USA.

[17] Xie BQ, Nancollas GH (2010). How to control the size and morphology of apatite nanocrystals in bone. Proc. Natl. Acad. Sci. USA.

[18] Davies E (2014). Citrate bridges between mineral platelets in bone. Proc. Natl. Acad. Sci. USA.

[19] Delgado-Lopez JM (2014). Crystal size, morphology, and growth mechanism in bio-inspired apatite nanocrystals. Adv. Funct. Mater..

[20] Wang Z (2016). Isoexergonic conformations of surface-bound citrate regulated bioinspired apatite nanocrystal growth. ACS Appl. Mater. Interfaces.

[21] Misra DN (1996). Interaction of citric acid with hydroxyapatite: Surface exchange of ions and precipitation of calcium citrate. J. Dent. Res..

[22] Kasemo B (2002). Biological surface science. Surf. Sci..

[23] Reid DG (2013). Citrate occurs widely in healthy and pathological apatitic biomineral: mineralized articular cartilage, and intimal atherosclerotic plaque and apatitic kidney stones. Calcified Tissue Int..

[24] Iafisco M (2015). The growth mechanism of apatite nanocrystals assisted by citrate: relevance to bone biomineralization. CrystEngComm.

[25] Delgado-Lopez JM (2012). Crystallization of bioinspired citrate-functionalized nanoapatite with tailored carbonate content. Acta Biomater..

[26] Sakhno Y, Ivanchenko P, Iafisco M, Tampieri A, Martra G (2015). A step toward control of the surface structure of biomimetic hydroxyapatite nanoparticles: effect of carboxylates on the {010} P-rich/Ca-rich facets ratio. J. Phys. Chem. C.

[27] Aina, V. *et al*. Surface sites of nanomaterials: investigation of local structures by *in situ* IR spectroscopy in Nanomaterials Imaging Techniques, Surface Studies, and Applications Vol. 146 (eds Fesenko, O., Yatsenko, L. & Brodin, M.) Ch. 12, 145 (Springer Proceedings in Physics, 2012).

[28] Bertinetti L (2007). Surface structure, hydration, and cationic sites of nanohydroxyapatite: UHR-TEM, IR, and microgravimetric studies. J. Phys. Chem. C.

[29] Sakhno Y (2010). Surface hydration and cationic sites of nanohydroxyapatites with amorphous or crystalline surfaces: a comparative study. J. Phys. Chem. C.

[30] Rey C, Combes C, Drouet C, Glimcher MJ (2009). Bone mineral: update on chemical composition and structure. Osteoporosis Int..

[31] Fleet ME (2009). Infrared spectra of carbonate apatites: ν(2)-region bands. Biomaterials3.

[32] Chen Y, Gu WJ, Pan HH, Jiang SQ, Tang RK (2014). Stabilizing amorphous calcium phosphate phase by citrate adsorption. CrystEngComm.

[33] Li CC (2011). Synthesis of citrate-stabilized hydrocolloids of hydroxyapatite through a novel two-stage method: A possible aggregates-breakdown mechanism of colloid formation. J. Colloid Interface Sci..

[34] Martins MA, Santos C, Almeida MM, Costa MEV (2008). Hydroxyapatite micro- and nanoparticles: nucleation and growth mechanisms in the presence of citrate species. J. Colloid Interface Sci..

[35] Nakamoto, K. *Infrared and Raman spectra of inorganic and coordination compounds*. Fourth Edition edn, 232 (Wiley, 1986).

[36] Geason, J., Jost, D., Merrill, P. H. & Caskey, D. Co-precipitated salts of fatty acids. Unated States patent (2011).

[37] Bolis V (2012). Coordination chemistry of Ca sites at the surface of nanosized hydroxyapatite: interaction with H2O and CO. Philos. Trans. R. Soc. A.

[38] Meldrum A, Wang LM, Ewing RC (1997). Electron-irradiation-induced phase segregation in crystalline and amorphous apatite: a TEM study. Am. Mineral..

[39] Bolis V, Busco C, Ugliengo P (2006). Thermodynamic study of water adsorption in high-silica zeolites. J. Phys. Chem. B.

