# Supplementary material for: On the surface effects of citrates on nano-apatites: evidence of a decreased hydrophilicity
Source: Sci Rep. 2017 Aug 21;7:8901. doi: 10.1038/s41598-017-09376-x (PMC5567200; doi:10.1038/s41598-017-09376-x)
Supplement: Supplementary file 1 — Supplementary Information [file 41598_2017_9376_MOESM1_ESM.pdf]

Supplementary Information

On the surface effects of citrates on nano-apatites: evidence of a decreased hydrophilicity

Pavlo Ivanchenko,[1] José Manuel Delgado-López,[1] Michele Iafisco,[3]
Jaime Gómez-Morales,[2] Anna Tampieri,[3] Gianmario Martra,[1]*Yuriy Sakhno[1]*

[1]Department of Chemistry and Interdepartmental Centre "Nanostructured Interfaces and Surfaces-NIS", University of Torino, Via P. Giuria 7, 10125 Torino, Italy.
[2]Laboratorio de Estudios Cristalográficos, IACT (CSIC-UGR), Avda. Las Palmeras 4, E-18100 Armilla, Granada, Spain.
[3]Institute of Science and Technology for Ceramics (ISTEC), National Research Council (CNR), Via Granarolo 64, 48018 Faenza (RA), Italy

Correspondence to gianmario.martra@unito.it; sakhnoyura@gmail.com

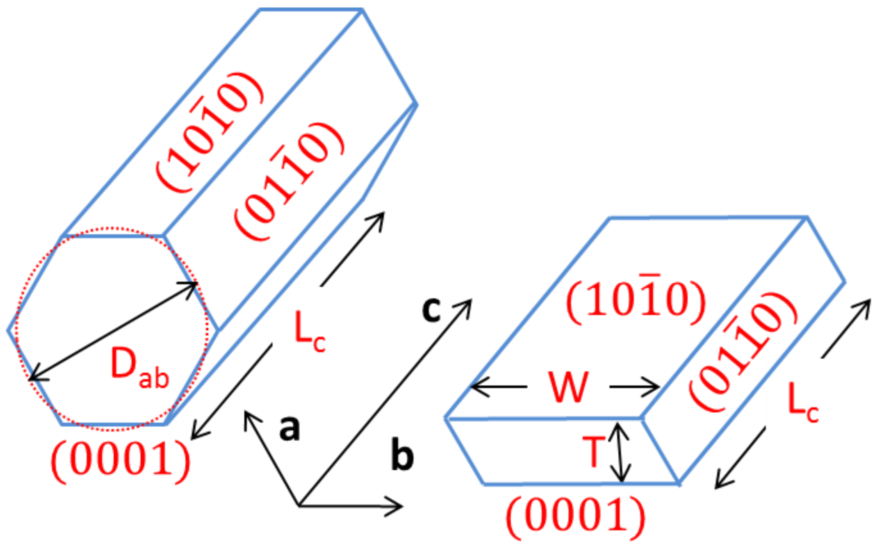

Supplementary Figure S1. Hexagonal (left) and platy (right) shapes of crystals used in ref. 1 for the Debye function analysis (DFA) of wide-angle X-ray total scattering (WAXTS) diffraction data collected for Cit-HA-4h and Cit-HA-96h. The most relevant (10$\bar{1}$0), (01$\bar{1}$0) and (0001) crystal facets are indicated. In the scheme on the left, D_{ab} is the diameter of the dotted circle, with an equivalent area of the hexagonal *ab* base. Adapted from ref. 1.

Reference

1. Delgado-Lopez, J. M. et al., Crystal size, morphology, and growth mechanism in bio-inspired apatite nanocrystals. *Adv. Funct. Mater.* **24**, 1090-1099, (2014).

Table S1. Summary of WAXTS-DFA, XRD, TEM and AFM characterizations (from ref.s 1-3, each quoted in all relevant points in the table) of Cit-HA samples. It includes:

- first section, with data on the relative amount of different phases/crystal morphology as by WAXTS-DFA: mass fractions of amorphous and crystalline phases (w_T %, where T= total), and mass fractions of crystalline hexagonal and platy shapes (w_C %, where C= crystalline);
- second section, with data on crystal domains: size of crystal domains estimated by applying the Scherrer equation to the 0002 and $31\bar{4}0$ reflections; size of crystal domains obtained by WAXTS-DFA (shapes and indexing of dimensions as in Figure S1): average dimensions D_{ab} and L_C (diameter of a circle of equivalent area of the *ab* base and the length along the ***c***-axis, respectively) and their standard deviations σ_{ab} and σ_c of hexagonal (rod) nanocrystals; average dimensions L_C, W and T representing the length along the *c*-axis, the width along the *b*-axis and the thickness of the *ab* base, respectively as well as their standard deviations (σ_c, σ_W, σ_T) of platy nanocrystals;
- third section with data on nanoparticles: length (L) and width (W) and their standard deviations (σ) of the 2D projections of nanoparticles on the TEM image plane; length (L) width (W) and thickness (T) and their standard deviations (σ) of nanoparticles as measured by AFM.

**Sample**	**Relative amount of different phases/crystal morphology (by WAXTS-DFA, ref.1)**	
	**Amorphous/Crystalline (w_T%)**	**Hex/Platy (w_C%)**
Cit-HA-4 h	11.6 / 88.4	43.4 / 56.6
Cit-HA-96 h	6.1 / 93.9	59.4 / 40.6

	**Dimensional and morphological insights (nm) From X-ray diffraction techniques: crystal domains**						
	**Crystal size by XRD (ref. 2)**		**Size of hexagonal crystals by WAXTS-DFA (ref. 1)**		**Size of platy crystals by WAXTS-DFA (ref. 1)**		
	**D_{0002}**	**$D_{31\text{-}40}$**	L_c (σ_c)	D_{ab}(σ_{ab})	L_c(σ_c)	W(σ_W)	T(σ_T)
Cit-HA-4 h	45.3	6.5	20.96 (9.28)	7.38 (1.02)	20.96 (9.28)	8.93 (1.37)	3.86 (0.59)
Cit-HA-24 h	48.0	8.6	-	-	-	-	-
Cit-HA-96 h	90.6	9.6	25.10 (11.94)	10.47 (1.83)	25.10 (11.94)	12.37 (2.46)	5.36 (1.07)

	**From microscopies: size of nanoparticles (nm)**						
	**TEM (ref. 2)**		**TEM (ref. 3)**		**AFM (ref.1)**		
	L (σ)	*W* (σ)	*L* (σ)	*W* (σ)	*L* (σ)	*W* (σ)	*T* (σ)
Cit-HA-4 h	84.8(16.2)	15.7(3.8)	29.3(9.5)*	6.2(1.7)	66.3(22.9)	38.9(15.1)	6.2(1.1)
Cit-HA-24 h	83.6(32.0)	13.7(6.1)	-	-	-	-	-
Cit-HA-96 h	104(43.2)	14.8(5.8)	44.1(17.8)**	7.6(1.6)	104.1(19.0)	56.6(12.2)	13.5(4.1)

* no projections of nanoparticles longer than 50 nm were observed

** ~ 15% of projection of nanoparticles 90-110 nm long were also observed

Supplementary Discussion S1

The Scherrer analysis of reflections in conventional XRD patterns and WAXTS-DFA provided significantly different values of the dimensions of crystal domains. As reported in the main text, WAXTS allow to take into account on the same footing both long-range order and short-range effects. Furthermore, the DFA approach (relying on the Debye-Scherrer equation) makes use of

calculated diffraction patterns from atomistic models of the crystals to reproduce the experimental pattern. Thus, WAXTS-DFA provides a more accurate and complete structural and morphological analysis, with quantitative information in terms of structure, composition, size distributions and morphology of nanocrystals.

In the case of HA nanocrystals mainly terminated by $\{10\bar{1}0\}$ planes, the determination of the size of the relevant $\{10\bar{1}0\}$ crystal domains by Scherrer analysis of the width of XRD reflections suffers of another limitation. Indeed, the $30\bar{3}0$ reflection is heavily overlapped to the $20\bar{2}2$ one and partially to the $11\bar{2}2$ reflection, (see Figure S4), which makes rather difficult the estimation of its width. To obtain information on a crystal domain size in a direction normal to the *c*-axis, the well-resolved $31\bar{4}0$ reflection is then usually considered.

From data in Table S1 it is also clear that dimensions of nanoparticles as measured by AFM (considered more reliable than TEM, *vide infra*) are significantly larger than crystal domains provided by WAXTS-DFA. In previous papers it was proposed that this discrepancy indicates that nanoparticles are the result of the attachment of primary nanocrystals.[1,3]

Differences in particle sizes obtained by TEM and AFM also deserve some comments. Particles observed by TEM have an arbitrary orientation on the sample grid, thus size and shape of their 2D projection on the image plane can differ from the actual ones. Indeed, different size distributions were obtained for both Cit–HA–4h and Cit–HA–96h when measuring the 2D projections in independent sets of TEM observations.[2,3] Conversely, in AFM measurements reported in ref. 1 and 3, less arbitrary sizes and shape of nanoparticles were imaged, because of the possibility to inspect them also along the vertical direction. This resulted in a better recognition of the orientation of nanoparticles on the support, and of a clearer imaging of individual nanoparticles also when part of agglomerates (see, in particular, the Electronic Supplementary Information of ref. 3).

Nevertheless, both TEM and AFM monitored the increase of the length of the main size of nanoparticles upon the 4-96 h maturation time.

References

1 Delgado-Lopez, J. M. et al. Crystal size, morphology, and growth mechanism in bio-inspired apatite nanocrystals. *Adv. Funct. Mater.* **24**, 1090-1099, (2014).

2 Delgado-Lopez, J. M. et al. Crystallization of bioinspired citrate-functionalized nanoapatite with tailored carbonate content. *Acta Biomater.* **8**, 3491-3499 (2012).

3 Iafisco, M. et al. The growth mechanism of apatite nanocrystals assisted by citrate: relevance to bone biomineralization. *CrystEngComm* **17**, 507-511 (2015).

Supplementary Discussion S2

***Calculation of the thickness of the amorphous phase supposed to be spread on the major faces (i.e. $\{10\bar{1}0\}$ ones) of Cit-HA nanoparticles*.**

Cit-HA with platy shape and sizes as measured by AFM carried out in ref. 1 were considered. The average length, $L(\sigma)$, width, $W(\sigma)$, and thickness, $T(\sigma)$ used are those listed in Table S1. The densities of HA (ρ_{HA}=3.156 g/cm^3) and amorphous calcium phosphate (ρ_{ACP}= 2.31 g/cm^3) have been collected from the literature (ref. 2 and 3, respectively).

For platy nanoparticles, the total volume can be determined from eq. 1

$$V_T = WLT = WL2a \qquad \text{where } T=2a \qquad [1]$$

and the increment of volume due to the amorphous layer assumed to be present, for the sake of simplicity, only on the two more extended $\{10\bar{1}0\}$ faces, from eq. 2

$$\Delta V = 2WL\Delta a \qquad [2]$$

In this formula Δa represents the thickness of the amorphous layer on each of the two $\{10\bar{1}0\}$ faces considered.

Δa can be determined from the equality:

$$\Delta a / a = (m_{ACP}/\rho_{ACP})/V_T$$

This equality is obtained by dividing eq. 2 by eq. 1 on one hand, and the term (m_{ACP}/ρ_{ACP}) with V_T in eq. 1, on the other hand.

The results of the calculations are listed in the table below:

Sample	wt% crystalline	wt% amorphous	Thickness (in nm) of the amorphous layer assumed to be present only of the two more extended $\{10\bar{1}0\}$ faces of platy nanoparticles
Cit –HA-4h	~90	~10	0.41 (0.14)
Cit –HA-96h	~94	~6	0.54 (0.16)

Hence, by considering also the other facets of nanoparticles as surfaces coated by the amorphous layer, and accounting on an average for ca. 20 % and 27 % of the total surface calculated for Cit–HA–4h and Cit–HA–96h, respectively, an even thinner amorphous layer should be present.

References

1. Delgado-Lopez, J. M. et al. Crystal size, morphology, and growth mechanism in bio-inspired apatite nanocrystals. *Adv. Funct. Mater.* **24**, 1090-1099, (2014).
2. Dorozhkin, S. V. Bioceramics of calcium orthophosphates. *Biomaterials* **31**, 1465-1485 (2010).
3. Holt, C., Timmins, P. A., Errington, N. & Leaver, J. A core-shell model of calcium phosphate nanoclusters stabilized by β-casein phosphopeptides, derived from sedimentation equilibrium and small-angle X-ray and neutron-scattering measurements. *Eur. J. Biochem.* **252**, 73-78 (1998).

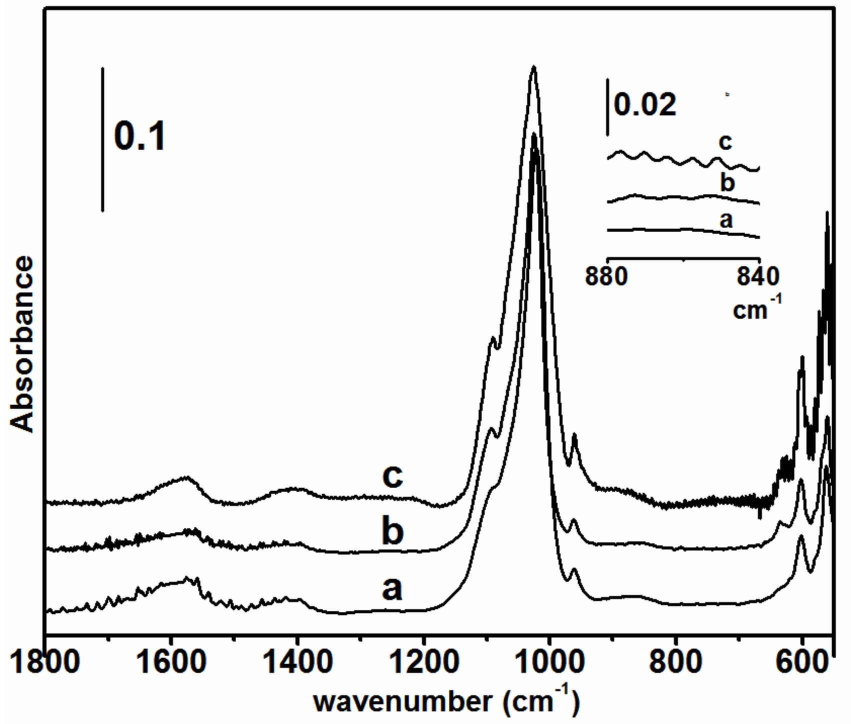

Supplementary Figure S2. IR-ATR spectra of: a) Cit–HA–4h, b) Cit–HA–24h and c) Cit–HA–96h. Inset: zoomed view of the 880-840 cm^{-1} range.

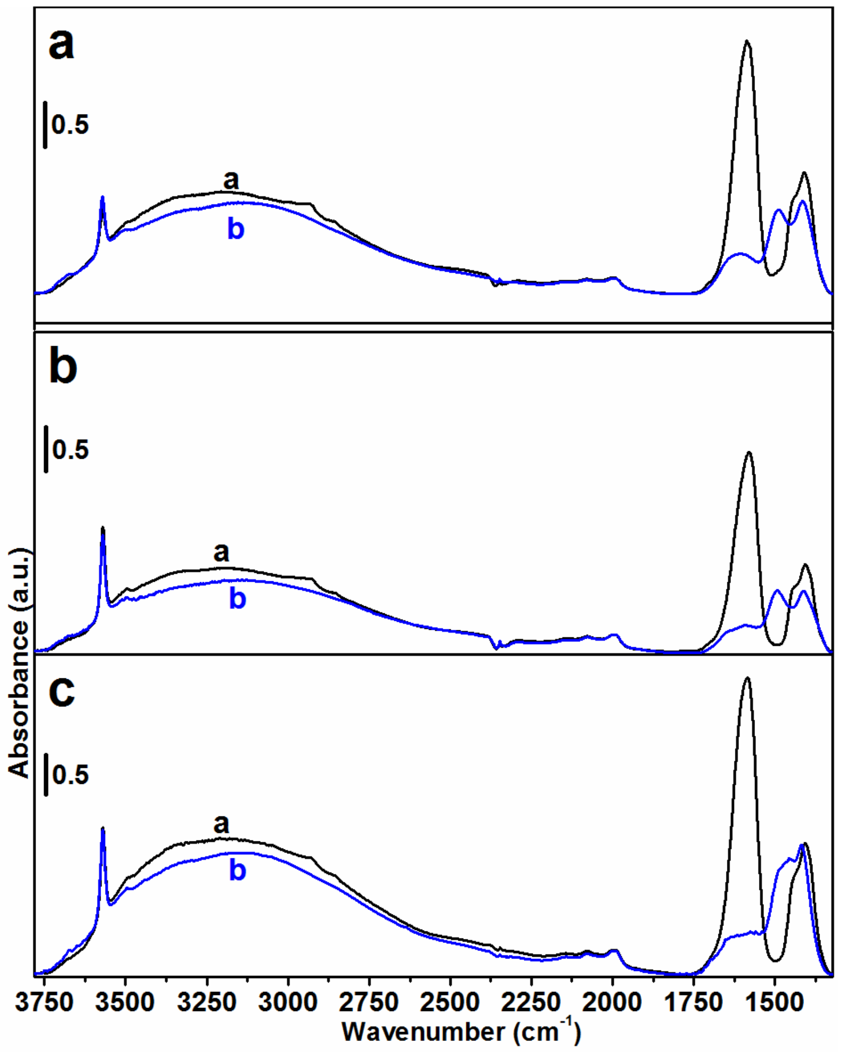

Supplementary Figure S3. IR spectra (transmission, self-supporting pellets) of **a**) Cit–HA–4h, **b**) Cit–HA–24h, and **c**) Cit–HA–96h after 60 min outgassing at b.t.: (a, black curves) in the presence of citrates and (b, blue curves) after their removal by washing with a 0.1 M NaOH solution.

Comment on Supplementary Figure S3

Bands due to the νCOO^- modes of citrates dominating the 1700-1300 cm^{-1} range of the pristine Cit–HA materials (black curves "a"), appeared almost depleted in the spectra of samples washed with the NaOH solution (blue curves "b"). Conversely, new bands due to carbonate groups appeared in this range.[1]

Reference

1. Fleet, M. E. Infrared spectra of carbonate apatites: ν(2)-region bands. *Biomaterials* **30**, 1473-1481, (2009).

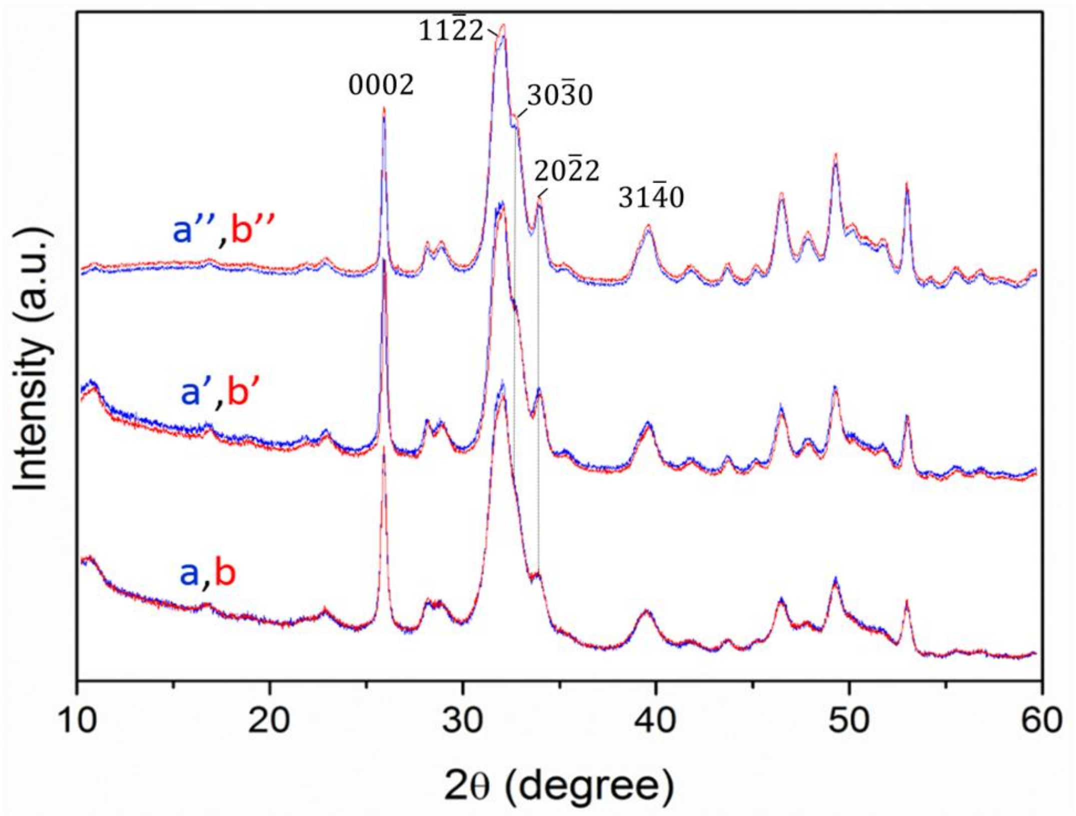

Supplementary Figure S4. XRD patterns of samples in their pristine form (blue curves) and after washing with a basic solution: a, b) Cit–HA–4h; a', b') Cit–HA–24h; a'', b'') Cit–HA–96h. The peaks correspond to the characteristic diffraction reflections of hydroxyapatite phase (JCPDS 9-432).

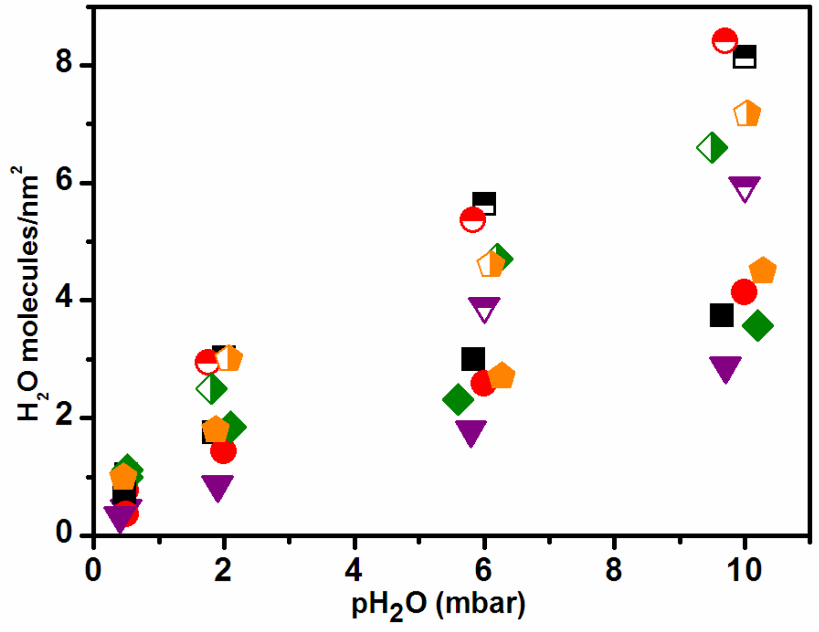

Supplementary Figure S5. Volumetric isotherms of H_2O vapour adsorbed at T= 303 K on the hydroxyapatite samples. Colour code of symbols is: **red**) HA1; **black**) HA2; **orange**) Cit-HA-4h; **purple**) Cit-HA-24h; **green**) Cit-HA-96h. Half-filled symbols: primary isotherm, i.e. admission of H_2O vapour on the HA samples outgassed at 433 K for 90 min. Full symbols: secondary isotherm, i.e. admission of H_2O vapour on HA samples resulting from the primary isotherm and then outgassed at 303 K for 120 min.
